# Efficacy of transcranial direct current stimulation for controlling of food craving in subjects with overweight or obesity

**DOI:** 10.1192/j.eurpsy.2024.418

**Published:** 2024-08-27

**Authors:** J. Kim, S. Yang

**Affiliations:** ^1^Psychiatry, Daegu Catholic University School of Medicine, Daegu, Korea, Republic Of

## Abstract

**Introduction:**

This study investigates the effects of transcranial direct current stimulation (tDCS) on food craving improvement and changes in brain function associated with craving in overweight and obese subjects.

**Objectives:**

Food craving disregards the homeostatic mechanisms related to appetite and nullifies the rewarding effects of food, directly contributing to body weight and eventually leading to obesity. In this study, we aim to explore the effects of transcranial direct current stimulation (tDCS) on food craving improvement and changes in brain function associated with craving by conducting a total of 10 sessions of tDCS over a period of 2 weeks on overweight and obese subjects.

**Methods:**

A total of 86 patients who were overweight or obese (BMI ≥ 23 kg/m2) during the study period were included. The tDCS montage involved placing the anode over the left and the cathode over the right DLPFC. Weight, BMI, neuropsychological variables, and food craving-related variables were assessed. We measured absolute and relative EEG power in 19 channels and analyzed QEEG according to the following frequency ranges: delta (1–4 Hz), theta (4–8 Hz), alpha (8–12 Hz), beta (12–25 Hz), high beta (25–30 Hz), and gamma (30–80 Hz).

**Results:**

After the application of tDCS, there was no significant reduction observed in weight and BMI. However, all measures related to food and eating showed a decrease in the intensity of cravings, and there was also a significant reduction in depression, anxiety, and perceived stress. In quantitative EEG analysis, an increase in theta waves was observed in the left frontal area (F7 and F3), an increase in alpha waves in the right parietal area (P4), and a decrease in beta waves in the frontal area (FP2) and occipital area (O1).

**Conclusions:**

This study investigated the effects of tDCS on food craving in overweight and obese individuals, and it was found that there were improvements in psychological factors such as depression and anxiety. Additionally, using quantitative EEG, neurophysiological changes were observed, including an increase in theta waves and a decrease in beta waves.

**Disclosure of Interest:**

None Declared

